# Cryptic Emotions and the Emergence of a Metatheory of Mind in Popular Filmmaking

**DOI:** 10.1111/cogs.12586

**Published:** 2018-01-22

**Authors:** James E. Cutting, Kacie L. Armstrong

**Affiliations:** ^1^ Department of Psychology Cornell University

**Keywords:** Conversations, Embodiment, Emotions, Faces, Mind reading, Movies, Theory of mind

## Abstract

Hollywood movies can be deeply engaging and easy to understand. To succeed in this manner, feature‐length movies employ many editing techniques with strong psychological underpinnings. We explore the origins and development of one of these, the reaction shot. This shot typically shows a single, unspeaking character with modest facial expression in response to an event or to the behavior or speech of another character. In a sample of movies from 1940 to 2010, we show that the prevalence of one type of these shots—which we call the cryptic reaction shot—has grown dramatically. These shots are designed to enhance viewers’ emotional involvement with characters. They depict a facial gesture that reflects a slightly negative and slightly aroused emotional state. Their use at the end of conversations, and typically at the end of scenes, helps to leave viewers in a state of speculation about what the character is thinking and what her thoughts may mean for the ongoing narrative.

One of the numerous strategies used by cultural representations to intensify [our] pleasure is to present our mind‐reading adaptations with fantasies of … complex social contexts in which people's bodies seem to provide direct access to their minds. —Lisa Zunshine (2012, p. 179)

## 1 Mind reading and the narrative arts

We recognize the brazenness of any title that lays claim to a metatheory of mind, but our goal here is relatively modest. We hope to demonstrate that Hollywood filmmakers have incrementally adopted a practice of promoting situations that encourage viewers to discern underlying emotional states from characters’ facial and bodily expressions. That is, we claim that filmmakers have developed a theory of viewers’ *theory of mind*. We discern the existence of that metatheory through the implementation of their craft. Moreover, filmmakers began this progression long before academia conceived of the concept (see Levin, Hymel, & Baker, 2013).

To begin, however, let us consider everyday life. A major daily task is to understand and respond to other people. Words matter a lot. We rely heavily on what others say, particularly in response to what we say. But more important in this context, we watch closely how people react to us and to the situations around us. That is, we discern and interpret how their bodies move, and we are particularly alert to how their faces respond. Beyond their words, we automatically read attitudes into their facial and body expressions and we draw conclusions, although not always correctly, about their mental states. Following the lead of many others (e.g., Nichols & Stich, 2003), we call this extra‐linguistic activity *mind reading*; Barrett (2017) has called it mental inference.

Mind reading might seem far removed from the narrative arts—particularly literature and cinema—but it is not (Levin et al., 2013; Oatley, 2011). Nor is it far from socialization processes that enrich us. Some of the educational and social benefits of reading fiction and of watching popular movies lie in the varied opportunities they provide us in understanding characters and in simulating their mental travails (Mar, Tackett, & Moore, 2010; Zunshine, 2006). These media often offer us rich benefits well beyond everyday contexts—within and across widely varying situations, within and across cultures, and across time (Oatley, 1999; Zunshine, 2006).^1^ Mind reading in fiction may even have been involved broadly in promoting human rights (Hunt, 2007).

In addition, more than real life, literature and film provide circumstances in which we can foster the agility of our mind‐reading abilities. That is, in novels and narrative movies, we get to find out more rapidly what characters actually thought about what happened in their diegetic (story) worlds,^2^ allowing us to test our predictive skills even in situations that may have been devised to be complex or exotic. In particular, as Zunshine (2012, p. 82) noted: “Once you start scrutinizing movies, you realize that … watching a movie is a rather extraordinary mind‐reading experience. … It seems that directors intuitively but persistently look for social contexts that allow characters to struggle to rein in their emotions.”

Again, this face/behavior/mind‐reading process is associated with what psychologists and philosophers have called a theory of mind—our attempts at understanding mental states of others (e.g., Carruthers & Smith, 1996). It is one implementation of our intentional stance toward others (Dennett, 1987); we assume that there are reasons that another person behaves and responds the way she does, and we try to figure out what they are. As Bering (2002, p. 12) noted, people “cannot turn off their mind‐reading skills even if they want to. All actions are … perceived to be the products of unobservable mental states.” And as Zunshine (2011, see also Barrett, 2017) suggested, this mind‐reading process is promiscuous, proactive, and periodically wrong.

In real life, of course, we do this mind reading of one another all the time. In literature, we read characters’ minds without actually seeing faces, although the description of body and facial movements is quite common (Zunshine, 2006). In movies, however, filmmakers deliberately show us the bodies and faces of characters, in part to allow us to assess any possible additional information that might be revealed through posture and facial expression.^3^

But if we say that the viewer is reading the mind of the character, what can we say that the character is “doing”? More precisely, how have authors and directors designed the narration so that readers and viewers are given an opportunity to try to discern the character's mental state? In cinema, this is often done best when the character is not talking. Why?

The major purposes of film conversations are to introduce characters and establish their goals, to create a network of diegetic relationships, and to provide information about plot‐turning events. Importantly, they also must not reveal too much too soon. In other words, at times conversations need to be subtle, often leaving things unsaid. Reaction shots do just that. If the emotion in the unspoken reaction is too clearly manifest, it may impair the dramatic impact of the later narrative. And perhaps most important, filmmakers want viewers to empathize with the protagonist—to understand her emotional responses to the buffeting of events around her. Her struggle *not* to speak suggests that something might have been said, and her lack of speech invites viewers to imagine what that might be. By withholding overt responses, filmmakers entice viewers into greater involvement with the characters.

For the construction of moments that signal mind‐reading opportunities, Zunshine (2012, p. 30ff) proposed three rules for an author or director to follow in revealing the character's thoughts and emotions.^4^ Her first is *contrast*, where in selected situations a focal character will become mind‐readable, others not. Thus, most characters most of the time will be generally unreadable beyond what they say. When a sole character in view is not speaking, however, viewers will pay more attention to her facial and bodily gestures, and this is when they invoke their mind‐reading skills to discern her emotional state. Second, the character's emotional state should be *transient*, coming and going quickly. And third, the physical expression of the character should be *restrained*, with her struggling to conceal her emotions. Indeed, it is the very visibility of the internal struggle that is the cue for the viewer to imagine what might have been said, and thus more intently try to discern her emotional state.

### 1.1 Goals, analytic path, and cultural assumptions

One goal of this article was to provide historical and empirical substance to Zunshine's scheme in the domain of popular movies. We do this by exploring how filmmakers have changed their style of presenting conversations over the course of the 20th century to enhance the participatory mental responses of viewers. Our exploration has three parts.

The first study explores *shot scale* (the size of a character's face within the movie frame) as it has changed since the beginning of feature‐length cinema. We will claim that technological advances (smaller and more mobile cameras) allowed filmmakers to more easily enlarge the image of faces for viewers. This trend makes facial and upper‐body gestures easier and more economical to discern. In other words, from a purely optical point of view, this change has fostered an increase in the viewer's ability to read the mental states of characters.

The second study looks at the representation of conversations and how they too have changed, with a focus on a particular class of reaction shots, which we call *cryptic reaction shots*. These are shots of a focal character who does not speak. We find such shots now end conversations much more often than they used to, whereas other types of reaction shots, which generally occur outside of conversations have decreased in a compensatory way. The first trend allows one character at a time (*contrasting* with the others) to appear briefly (*transiently*) discomfited, perplexed, or overwhelmed such that she appears to struggle not to speak (is *restrained*), perhaps not knowing what to say.

And the third study is an experiment on viewers’ assessment of facial emotions seen in stills taken from cryptic reaction shots. Results show that these expressions are, for the most part, slightly negative and reveal modest arousal. We suggest that the slight negativity means that the character has not completely accepted the ending premises of the conversation, and that the modest arousal means that she is stifling an urge to speak. Thus, we propose that these attributes of emotional response—slight negativity and slight arousal—jointly provide fertile ground for actively imputing emotional states.

We conclude, with backup from theory in film and literature studies, that the expressions in shots at the end of conversations are designed to engage the mind‐reading capacities of viewers. We make no claim that these expressions necessarily represent a universal or biological predisposition. Instead, we embrace the worldwide penetration of Hollywood film.^5^ These reaction shots project to viewers what Hollywood filmmakers have assumed will promote further engagement. This assumption is corroborated in judgments of stills by a convenience sample of viewers who are members of the central target audience. We also make no claim that the expressions seen in these movies by viewers from different cultures would inherently be the same as in their native societies. We would claim, however, that viewers, because of their exposure to Hollywood movies, would recognize a character's struggle not to speak, and that this recognition would trigger in the viewer a desire to know more about what the character is thinking.

## 2 Windows on a silent character's mind: Four types of reaction shot

The basic unit of a movie is the *shot*. A shot is a continuous run of motion in frames taken by a camera (or composed by an animation system) segregated from the previous and next shot by a cut, dissolve, fade, or some other transition. In contemporary films, most shots last between 2 and 10 s, and there are typically between 1,500 and 3,000 of them. Older movies have longer and fewer shots. Shots can be classified into a dozen or more categories (e.g., Cutting & Candan, 2015), but the core opportunity for viewers to exercise their mind‐reading abilities in cinema occurs with the *reaction shot*. This is a shot of a character who responds silently to an event or a person in the diegetic world. Such an event is typically shown in a previous *point‐of‐view* (POV) shot—from the standpoint of the character—and the shot of the character will follow, but occasionally precede, that POV.^6^

Cinematographer Brian Brown (2012, pp. 23–24) noted that: “Reaction shots are very important … Silent films were the apex of reaction shots as a method … it is when you see the facial and body language reactions of the listener that you get the entire emotional content of the scene.” Film historian David Bordwell (2008) elaborated: “In the 1910s, directors began systematically creating a scene from separate shots … In this approach, particularly as practiced in Hollywood, a person's facial expression could become part of an ongoing suite of shots, each concentrating on one item of information. Thanks to cutting, the facial reaction could be underscored, sharpened, and timed for best effect.” But despite Brown's (2012) comment about its silent‐film apex, reaction shots have hardly gone away. Thus, it is worthwhile to distinguish four types of reaction shot found in contemporary media.

### 2.1 The noddy

The first reaction shot stems from a description of British television interviews (Fiske, 1987). Most of an interview may feature long‐duration shots of the interviewee, but there will be occasional, interspersed, short shots of the interviewer, often nodding about what has been said. The noddy has an important counterpart in real‐life conversation. In real life, such responses by the listener are sometimes called co‐narration; they are information for the speaker as to whether or not the listener has followed what was said (Bavelas, Coates, & Johnson, 2000). The inclusion of the noddy in televised interviews may be to make it seem like a more natural conversation to the viewer and to act as an endorsement of what was said. Nonetheless, the noddy is not typically part of cinema; movie conversations are not typically interviews.

### 2.2 The commentary reaction shot

**Figure 1:**
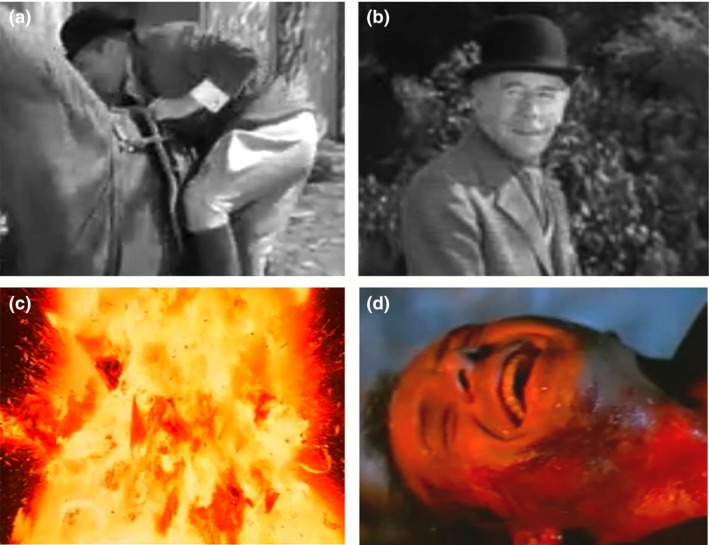
Reaction shots and their antecedent point‐of‐view (POV) shots. In our terminology, the still in panel (b) is a *commentary* reaction shot, and that in panel (d) is an *open‐response* reaction shot. Panels (a) and (b) are details from *The Philadelphia Story* (1940), where George Kittredge (John Howard) scrambles to get on horseback and, in the commentary shot, Uncle Willie (Roland Young) smirks at his ineptness. This reaction tells the viewer to interpret Kittredge as an outclassed interloper. Panels (c) and (d) are details from *Die Hard 2* (1990), where John McClane (Bruce Willis) reacts to the explosion of a plane full of international criminals. McClane's response is, given the context of the movie, entirely predictable and is openly readable as joy. Examples of another type, which we call the *cryptic* reaction shot, are shown in Fig. 4.

The purpose of this second type of reaction shot is to comment silently on an event that just happened. For example, philosopher Nöel Carroll (2010, p. 298) noted: “We know when a character has done something very stupid, even if it is not immediately obvious, because there is a reaction shot of someone else looking contemptuous.” These shots no longer seem common in popular cinema—perhaps because they are not very subtle—but one is shown in Fig. 1b taken from *The Philadelphia Story* (1940). George Kittredge (John Howard) scrambles to get on horseback (Fig. 1a), and in the subsequent reaction shot, Uncle Willie (Roland Young) quietly smirks at his ineptness. This is our first indication in the movie that Kittredge—fiancé to Tracy Lord, the female focus of the narrative—is not her social equal. He is new money and demonstrably maladroit; she and the others are old money and adept within their social advantages.

The commentary shot has an historical analog in Renaissance painting. In the 15th century, Leon Battista Alberti wrote about his endorsement of certain large, complex narrative artworks: “In an *istoria,* I like to see someone who admonishes and points out to us what is happening there” (Spencer, 1956, p. 78). In other words, there should be a commentator within the painting whose gesture tells us how to interpret an event.

### 2.3 The open‐response reaction shot

In this third type of reaction shot, the character's responses are mostly predictable, entirely understandable, and eminently readable. Emotions are not veiled. For example, in *Die Hard 2* (1990), John McClane (Bruce Willis), shown in Fig. 1d, has just set fire to an airplane full of international criminals. He reacts to its explosion (seen in the POV shot, Fig. 1c) in relieved laughter after a complex network of prior tribulations. McClane speaks in the shot, but he need not have; no one else is on the tarmac to hear him. Open‐response reactions seem to follow a long buildup to a complex situation where tension has reached a maximum and is suddenly released, as in the end of a climax.

### 2.4 The cryptic reaction shot

Finally, the fourth has strong ties with Zunshine's notions of contrast, transience, and restraint. It is the centerpiece of this article, and it is the type of shot that is most relevant to mind reading in our context. Here, we constrain the empirical scope of this shot to situations within conversations where a focal character says nothing. This stipulation is important; otherwise, the shot would simply be part of the ongoing sequence of characters talking. This type of shot can, in principle, be inserted anywhere into the conversational sequence. Indeed, our analyses show that 75% of them occur in mid‐conversation, but that 88% of these conversations also end with a reaction shot. For our purposes, it is the placement of a cryptic reaction shot at a conversation's end that is critical.

## 3 Study 1: Shot scale and gaze congruence

This initial study poses two questions. First, have there been systematic changes in mean shot scale over time? And second, if found, what is the purpose of this change?

### 3.1 Faces and shot scale

Unsurprisingly, the most important visual objects for people—in the real world and in cinema—are human (or character) faces. Evidence for the former, if any be needed, includes the facts that newborns prefer faces to other objects (Fantz, 1961; Farzin, Hou, & Norcia, 2012), and that Internet surfers on Instagram “like” pictures with faces 38% more than those without, and are 32% more likely to write further comments on them (Georgia Institute of Technology, 2014). Evidence for the latter stems from the fact that 90% of all shots in popular movies show the face of at least one character, and that this has been quite constant across the history of popular cinema (Cutting, 2015).

**Figure 2:**
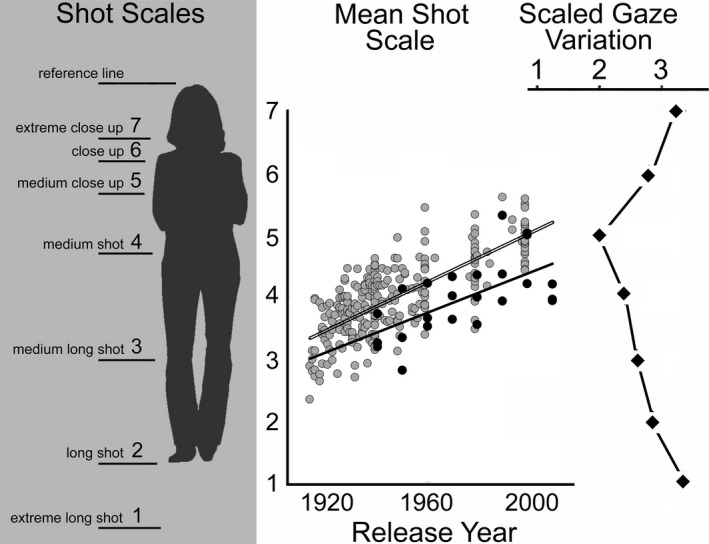
The left panel shows a mock‐up of how shot scales are determined and numbered (adapted from Cutting & Armstrong, 2016). The central panel shows the results of Study 1. The gray dots are the mean shot scales of 271 movies analyzed by Salt (1992, 2006, and http://www.cinemetrics.lv/satltdb.php#ss). The black dots are the mean shot scales for 24 movies analyzed by Cutting et al. (2012). The right panel shows, as diamonds, the data of Smith (2013; Mital et al. 2011) for the variability across viewers of their fixation position on personal screens as a function of shot scale. In this display, leftward shifts indicate more uniform gaze across viewers (less variation) and rightward shifts less uniformity (more variation). Because theater screens take up more of the visual field, the function for theater viewing would be shifted somewhat downward. Either way, notice that the convergence of the mean shot scale data for movies and uniformity of cross‐viewer gaze fixation as a function of shot scale. The data plots are an extensive elaboration and reconfiguration of Cutting (2015, Fig. 5).

*Shot scale* can be thought of as the measure of the size of the focal face within the movie frame. Variation in shot scale stems from opposite cinematic needs—assessing how important is it to see the environment, and how important is it to see a character's face. Shot scale is typically broken into seven categories. For purposes of later computation, we will rank order these, 1 through 7, corresponding to shots with visually small heads to visually large heads, respectively. Consider a standard terminology for the seven types of shots (Bordwell & Thompson, 2004; but see Salt, 1992; p. 142, and Smith, 2013; for some variation) and shown in the left panel of Fig. 2. In (1) an extreme long shot, the character does not fill the vertical extent of the screen; in (2) a long shot, the character's head and feet generally delimit the height of the screen; in (3) a medium long shot, the character is cut off at the knees; in (4) a medium shot, she is cut off at the waist; in (5) a medium close‐up at mid‐chest; in (6) a close‐up at the shoulders; and in (7) an extreme close‐up, only the head or part of the head might be shown. However, shot scale is truly continuous and its measure invites a certain amount of subjectivity. It can change across a shot as the camera and/or character move. In our analyses, we have marked the scale at the beginning of each shot (Cutting, Brunick, & Candan, 2012).

### 3.2 Movie sources and methods

We employed two sources of data for shot scale measurements in movies. No films were members of both sets. For each movie, mean shot scale is determined in a quantitative way, by multiplying the rank of the shot category's scale (1 to 7) by the frequency of its occurrence, then dividing the result by the number of shots. The first source is the sample of 24 popular Hollywood movies studied by Cutting et al. (2012, which lists the films in its appendix)—three per year (one drama, one comedy, and one action film) released from 1940 to 2010 at 10‐year intervals. Each film was among the most popular of its release year. Cutting et al. categorized the scale of every shot in each movie. Each had also been analyzed for its various shot types and shot frequencies (Cutting, 2014), as well as its scene structure (Cutting, 2014; Cutting et al., 2012).

Second, over the years, Barry Salt has provided shot distributions of older and of more contemporary films in his books (Salt, 1992, 2006) and has continued to offer these and new analyses online (http://www.cinemetrics.lv/satltdb.php#ss). As of April 22, 2017, Salt had produced such distributions for 271 different English‐language movies released between 1913 and 1999. However, while he acknowledged the simple calculation above, he never carried out a computational analysis looking at even a portion of the set.

Finally, here and in the following two studies, we report post hoc two‐tailed power (π) analyses for *p* values where π < 0.99.

### 3.3 Results

The mean shot scales for each of these movies are shown in the central panel of Fig. 2. The data for Salt's analyses are shown as gray dots with a black surround, and the overlapping data of Cutting et al. (2012) are shown as black dots. Consider the data based on Salt's analyses first. Notice that, although there are chronological gaps in his samples, there is a striking linear trend (*t*(269) = 18.4, *p *<* *.0001, *d *=* *2.25), indicating a shift from near a medium long shot to a medium close‐up over 86 years. Indeed, from his data, the regression‐equation value for the year‐2000 shot scale is 5.01, almost exactly a medium close‐up.

The data based on the analyses of Cutting et al. (2012) show a similar linear trend (*t*(22) = 3.73, *p *<* *.002, *d *=* *1.67, π = .80), but with values slightly shifted toward longer‐scaled shots. This trend's year‐2000 regression value would be 4.38. Some variation across measurers is to be expected, and some of this difference may be due to the fact that Salt considered a medium close‐up to be a shot from the waist up (Salt, 1992; p. 142), rather than the chest up (Bordwell & Thompson, 2004; Smith, 2013). Regardless, across both data sets, one can see that the mean movie shot has gradually moved toward a medium close‐up. Why?

One reason is technological and corresponds to the gradual decrease in size and accompanying increase in mobility of movie cameras. A second is psychological and pertains to how filmmakers have increasingly learned to control the gaze of the viewer. Smith (2013, p. 182) has shown that, under viewing conditions that mimic watching a movie on a high‐definition television (HDTV) or personal computer screen, a medium close‐up (chest up) typically shows the head of the character such that it minimizes the variability in fixation positions of viewers. Smith's data, reassembled from those of Mital, Smith, Hill, and Henderson (2011), are redrawn in the right panel of Fig. 2, aligned with the shot scales of the central panel, and ratio‐scaled as a function of gaze‐cluster covariance. Here, the smaller the number is, the lesser the spatial variation there is across different viewers’ fixation positions. Notice that the minimum variation in gaze fixation across viewers occurs with the medium close‐up.

Optically, theater screens take up considerably more of our visual field (~40° in width) than those of HDTVs or laptops (~20°), so to be more appropriate for viewing scales in a theater, Smith's function should be shifted down a bit, probably having a minimal gaze variation near a medium shot. Nonetheless, as <10% of contemporary movie watching currently occurs in theaters (British Film Institute, 2012), Smith's measurements are appropriate for most viewing contexts.

### 3.4 Discussion

The eyes and mouth are the centers of visual information for facial affect. This may seem obvious today, but it was not always so. In the 17th century, the French painter and art theorist Charles Le Brun was convinced that eyebrows were the main conveyors of affect (Montagu, 1994). However, Schurgin et al. (2014) showed that, in judgments of emotion in extreme close‐ups of static faces (requiring multiple fixations), 35% of all fixations were on the eyes, and 29% were on the lower nose or lips, but <4% were on the eyebrows.

In a medium close‐up shown on a typical personal screen, the viewer tends to look near the dorsum (middle) of a character's nose (Võ, Smith, Mital, & Henderson, 2012) and does so because relatively high acuity can be simultaneously obtained for both the character's eyes and mouth. A shorter‐scaled shot (close‐up or extreme close‐up) will move the eyes and mouth farther apart in the image, and the viewer must choose which, the eyes or the mouth, to look at. A longer‐scaled shot, on the other hand, renders the face smaller and makes facial expression more difficult to discern (Cutting & Armstrong, 2016). The shoulders are also shown in a medium close‐up, and these can be important to emotion assessment. Aviezer, Trope, and Todorov (2012) showed that body posture carries important information about the state of arousal of an individual.

Thus, there is an important sense in which, in standard movie viewing situations, the medium close‐up is the optimal shot. It allows filmmakers to exercise the most control over the gaze position of viewers. By decreasing variance in gaze, they give an increasing number of viewers the same, and the intended, visual experience. We propose that, with changes in technology (particularly smaller cameras), filmmakers have exploited their newfound mobility and created a film style that minimizes the effort of the viewer in picking up emotional information from the face. Moreover, the data of Study 1 show that, whether done so consciously or not, filmmakers have composed their works in just this manner.

## 4 Study 2: How conversations end in popular cinema

The results of Study 1 suggest that the style of popular movies evolved, in part, to show characters’ faces more clearly. Why? The core parts of popular cinema are its conversations (Bordwell, 2005, p. 22), and the mean shot scale of conversation shots is almost exactly the mean shot scale of an entire movie (Cutting, 2014), highlighting the pertinence of the results shown in Fig. 2.

The major questions addressed in this second study are: How do popular movie conversations end? What is the purpose of these ending shots? Moreover, have there been changes over time? Two other queries are also of interest. First, Salt (2006, p. 321) suggested that there has been an increase in reaction shots in American movies, so that too will be investigated. Second, Zunshine's (2011, 2012) second rule is *transience*. One way for filmmakers to create brevity in this window of transparency would be to make a reaction shot briefer than the other shots in the conversation of which there are many kinds. Thus, it is prudent to briefly discuss the others.

### 4.1 Other shot types in filmed conversations

The most basic conversation shot is the *master shot*, also called the *establishing shot*. These show all the potential conversants (two to many) and their arrangement in an environment. Many master shots occur before a conversation actually begins.

The most common conversation shots, however, are those that alternate presentation of the speakers. These come in two forms. The first is the *shot/reverse‐shot* combination (SRS, also called reverse‐angle shots) where an initial shot shows only one stationary conversant, followed by a shot of the other stationary conversant, and successive alternations between the two. Typically, these are staged and filmed such that one character is facing to the right and occupies the center‐left of the screen and the other faces left and occupies the center‐right of the screen. The second type is a variant on the SRS, which we consider separately here, called the *over‐the‐shoulder* (OTS) shot. Here, the spatial relations are similar, but the camera image includes the shoulder, head, and/or body of one character (typically out of focus) facing away from the camera and toward the other character (in focus and typically seen in three‐quarter profile).

### 4.2 The cryptic reaction shot

Again, the cryptic reaction shot shows a single character in conversation, but she does not talk. Instead, she is either listening to the other conversant or reacting to the conversation's end. Notice that the categorization of a shot as a reaction shot supersedes its potential categorization as an SRS or an OTS shot.

The mean scale of conversation‐ending reaction shots across the 24‐movie sample is 4.2, close to a medium shot. But since shots have progressed toward close‐ups over the years, the scale of reaction shots from 1990 to 2010 is 4.4, about halfway between a medium shot and a medium close‐up. Such results seem optimal for some combination of both theater and personal screen viewing (HDTV and laptop), following the results of Study 1. Filmmakers generally give viewers a glimpse of a nonspeaking character that is nearly ideal for gathering information from the eyes, from the mouth, and also from the shoulders and upper arms.

### 4.3 Methods for selecting movie conversations

We again employed the 24 US‐made movies selected by Cutting et al. (2012) and explored as part of Study 1. Across the roughly 31,000 shots in these movies fully 65% of them depict part of a conversation (Cutting & Candan, 2015), although this percentage varies by genre (77% in dramas, 69% in comedies, and 49% in action films). From these movies and working with previous data, the first author re‐watched them and selected all conversations that met the following criteria: (a) two and only two characters spoke, (b) one character spoke at least twice and the other at least once, and (c) it contained at least four shots but could, provided it had more than four shots, contain extra‐dialog shots within the sequence, such as POVs, inserts, or cutaways.

It also seemed prudent to exclude some conversations, even though they met these criteria—those involving any nonhumans or masked humans (*Beneath the Planet of the Apes,* 1970; *The Empire Strikes Back,* 1980) for whom facial gestures are difficult or impossible to discern; and those including voiceovers revealing the verbal thoughts of the characters who, in the diegetic world, were not actually speaking (*Goodfellas,* 1990; *What Women Want,* 2000).

On the basis of these criteria, these movies had between 7 (*Harvey,* 1950; *Inherit the Wind,* 1960) and 64 and 66 (*Valentine's Day,* 2010; and *Inception,* 2010) such conversations, for an overall total of 679. There is no difference in the number of two‐character conversations across genres (dramas, 27.5; comedies, 26.3; action films, 31.3). However, as the above limits suggest, there is an increasing number of such conversations across release years (*t*(22) = 7.98, *p *<* *.0001, *d *=* *3.4), with 13.3, 13.7, 35.0, and 51.3 in the four two‐decade spans (40s–50s, 60s–70s, 80s–90s, and 00s–10s), respectively. Because of this trend, our data below are reported in terms of proportions, rather than raw numbers, of two‐character conversations per film that end with a cryptic reaction shot.

The increase in the frequency of two‐character conversations is entirely in line with two other factors. The first is the increasing number of shots per film across this sample (and corresponding decrease in average shot duration), from around 500 in the 1940s and 1950s to as much as 2,000 and 3,000 in the most recent two decades. Our four‐shot criterion means that shorter shot durations allow for the inclusion of many more brief conversations. Indeed, the two‐character conversations with a sufficient number of shots to be included here have become briefer across release years (*t*(677) = −7.36, *p *<* *.0001, *d *=* *0.56). The data by decade are noisy but prior to 1975 they averaged 104 s, whereas afterward, they averaged only 60 s.

Second, there has been a marked decrease in the number of characters appearing together onscreen (Cutting, 2015). Films from the 1940s and 1950s averaged 2.5 characters per frame, whereas those of the 1990s and 2000s averaged only 1.5 per frame. This factor is a correlate of the fact that older films have more conversations among three and more characters, which because of their relative complexity of staging and camera coverage are excluded here.

### 4.4 Results

Of the 679 two‐character conversations, 300 ended with at least one cryptic reaction shot, and sometimes with more. Of these, across genres, there were 111 in dramas, 81 in comedies, and 108 in action movies; and across the three movies in the eight decades, there were 6, 10, 6, 9, 43, 58, 78, and 90, respectively, from 1940 to 2010. But remember, given the increase in number of two‐person conversations across years and their decrease in duration, we report below the proportions of conversations ending with cryptic reaction shots, not raw values.

**Figure 3:**
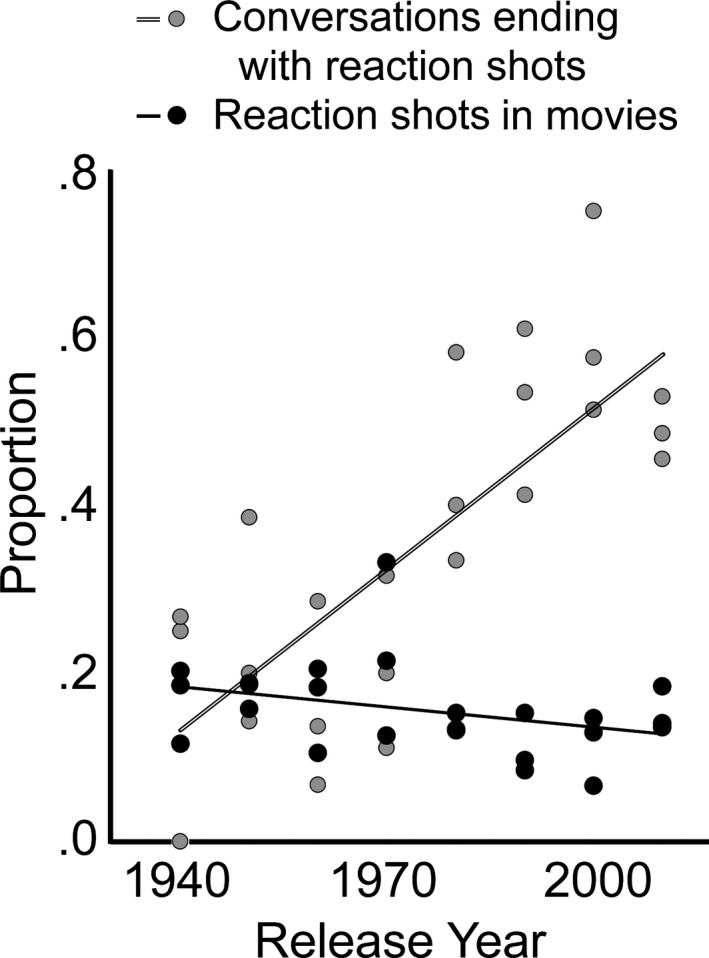
The results of Study 2. The rising function shows the proportion of all two‐character conversations in 24 movies that end with cryptic reaction shots (gray filled dots) and the relatively flat function shows the proportion of all reaction shots (black dots)—cryptic, commentary, and open response—as a function of all shots in a movie.

Figure 3 overlays two functions related to these data. The rising regression line is a plot, by release year, of the proportion of conversations in the 24 movies (shown as gray dots) that end with a reaction shot. Clearly, these have increased substantially from 1940 to 2010 (*t*(22) = 5.6, *p *<* *.0001, *d *=* *2.39, π =0 .88). The relatively flat function depicts the proportion of all shots in each movie (shown as black dots) that are reaction shots of any kind. This function dips slightly over time, but without import. Its flatness denotes that, although the absolute number of reaction shots has certainly increased in popular movies, as Salt (2006) suggested their proportion has not. Moreover, in the context of Zunshine's (2012) rules, their proportion here (about 17%) seems sufficiently uncommon (*contrasting* in her terms) to fit within her scheme.

Given the proportional constancy of reaction shots in movies over 70 years, the difference between these functions can be taken as a measure of the growth of cryptic reaction shots at the expense of commentary and open‐response reaction shots. Importantly, the two functions in Fig. 3 reveal an interaction (*t*(22) = 5.2, *p *<* *.0001, *d *=* *2.21, π = 0.78). The difference also shows the growing importance to filmmakers of cryptic reactions as mind‐reading seeds for viewers’ anticipation of narrative development.

In this manner, whereas reaction shots have been a part of the repertoire of filmmakers since the silent era (Bordwell, 2008; Brown, 2012; Thompson, 1985), their roles have been gradually reshaped. They have always served to indicate the reaction of a character to an event or to something that has been said, but over the years, they have been systematically appropriated into conversations, particularly as a device to end them, leaving the viewer to contemplate the implications of the conversation for the last‐seen character.

In keeping with Zunshine's (2012) notion that glimpses into the mental states of characters should be transient, within‐movie comparisons of shot durations are prudent. The mean duration of reaction shots in these films is 3.4 s, whereas that for SRSs and OTS shots is 5.6 s. Indeed, all 24 movies had reaction shots shorter than these conversation shots (*z *=* *4.79). Conversation‐ending reaction shots, more particularly, tend to be a little longer (4.3 s), but even these are shorter than the other conversation shots (*t*(19) = 3.24, *p *<* *.004, *d *=* *1.49, π = 0.54). As cryptic reactions also tend to end scenes, their brevity goes strikingly against the more general trend that final shots of scenes tend to be longer in duration than all the shots that precede them except the first (Cutting et al., 2012).

Finally, among the 300 conversations that end with reaction shots, 55 ended with multiple tokens; 37 ended with two consecutive reaction shots, 13 ended with three, 4 with four, and 1 with six. That is, the restrained characters looked silently back and forth at one another in successive shots. Perhaps unsurprisingly, these often happen at highly emotional, plot‐turning points in the narrative. We return to this idea in the next section.

There seems to be little limit on how many cryptic reaction shots can be concatenated to end a conversation. The possible leader in this domain is a film outside this sample, *Meet Joe Black* (1998). Fairly near the beginning of the movie there is a coffee‐shop conversation between a then‐unnamed fellow (Brad Pitt) and Susan Parrish (Claire Forlani). She is the daughter of a rich business man and at the time is engaged to his top assistant. The flirtatious but relationship‐quashing conversation ends with 12 reaction shots, oscillating back and forth between each character failing to hail the other to reconsider. Pointedly, the fellow is then immediately killed in a traffic accident.

### 4.5 Another account

But perhaps the cryptic reaction shots are not signals for mind reading by viewers, but simply a matter of plot progression. They might be signals of the character's reflection on, or dissatisfaction with, events in the flow of the narrative. If so, this might suggest that there would be a change in the frequency, perhaps a decline, of cryptic reaction shots over the course of the narrative as it approaches its dénouement.

**Figure 4:**
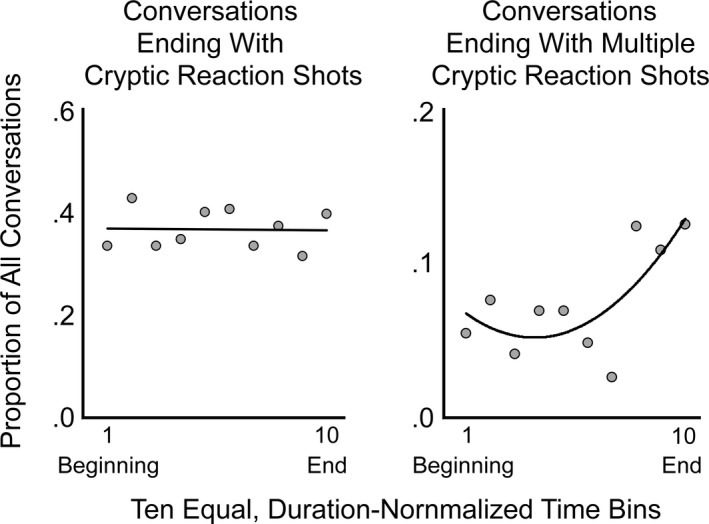
The left panel shows the proportion of two‐character conversations ending cryptic reaction shots across 10 equal‐duration time bins normalized for each of the 24 movies. The right panel shows the proportion of those conversations ending in multiple cryptic reaction shots (2, 3, or more) across the same time bins.

To test this idea, we divided each of the 24 movies into 10 equal‐duration bins and considered all the relevant conversations in each bin. We then counted the number of conversations ending with at least one reaction shot and divided these by the bin's total number of conversations. The resulting pattern is shown in the left panel of Fig. 4. Clearly, there is no trend (*r *=* *−.027, JZS Bayes factor = 1.002), and it is safe to assume that there is no change in terminal cryptic reaction shots across the duration of movies. Thus, such shots can have nothing inherently to do with the progression of the larger narrative.

However, if one uses the temporal bins to predict the proportion of conversations that end with more than one cryptic reaction shot (2, 3, or more), the result is quite different, as suggested in the right panel of Fig. 4. The first seven bins show relatively few conversations ending with multiple cryptic reaction shots, but the last three bins show considerably more. Although it may not be the best function to choose from, we fit these data with a second‐order polynomial. The correlation is quite striking (*r *=* *.74, *t*(8) = 3.15, *p *<* *.014, *d *=* *2.22, π = 0.67). Clearly, the last three‐tenths of movies, which consist mostly of the climax (Cutting, 2016; Thompson, 1999), appear to have conversations that invite the viewer to assess the mental and emotional states of both characters.

### 4.6 Discussion

Study 1 showed that the mean shot scale of popular movies increased over the 20th century, converging on one between a medium shot and a medium close‐up. Study 2 showed that, over the last half of the 20th century and into the 21st, an increasing proportion of conversations in movies end with a reaction shot of that same scale. The cryptic reaction shot, in which the last‐seen character does not speak, is an excellent vehicle for viewers to extract some additional emotional content in the facial expression. Indeed, this content may be part of the *dangling cause* (Bordwell, 2006), information for the viewer at the end of a scene to be picked up later in the movie. Such causes drive forward, scene by scene, the emotional substrate of the narrative.

Perhaps most strikingly, however, we know of no treatment of reaction shots in the film literature that shows any awareness of the change in their use over the 20th century. Clearly, some film scholars have been alert to some of the functions of reaction shots and their tie to emotional states (Bordwell, 2008; Brown, 2012), but among all the documented changes in movies, the functions and historical changes of reaction shots have not been a part of them.

Study 3 is an experiment that assesses the nature of the perceived expressions in cryptic reaction shots. The major question is: Just how *cryptic* is the emotion revealed in these shots? The potency of facial expression in a reaction shot rests on its relatively neutral tone, allowing some complexity to be read into the cinematic context by the viewer. Indeed, generally neutral facial expressions are known to elicit orienting responses (i.e., a furrowed brow, heart rate deceleration) in perceivers, likely as the result of perceivers attempting to decode the emotional content of these faces (Vrana & Gross, 2004). This orienting response may be due to the fact that the amygdala, which has been described as a relevance detector, is activated in response to neutral faces (Sander, Grafman, & Zalla, 2003), suggesting that neutral expressions invite mental inferences about them. In addition, Carroll and Russell (1997) showed that facial expressions in Hollywood movies were a fruitful domain of investigation in assessing emotional responses.

## 5 Study 3: Judging emotions in stills taken from cryptic reaction shots

Perhaps surprisingly, facial emotions are *not* better judged from dynamic displays than from static ones (Fiorentini & Viviani, 2011; Gold et al., 2013; but see also Ambady, Schooler, & Cohn, 2005). One reason for a lack of superiority in their recognition is that viewer responses are more variable (Hehman, Flake, & Freeman, 2015). Moreover, as most experiments in the emotion literature have used static faces, we will do so here as well, taking stills out of the cryptic reaction shots.

Emotions can be classified in several ways. We prefer to base viewer assessments on the outlines of the circumplex model (Posner, Russell, & Peterson, 2005; Russell, 1980). This model has two dimensions of emotion—valence (positive vs. negative emotional expression) and arousal (the intensity of the physical expression).

### 5.1 Stimuli

**Figure 5:**
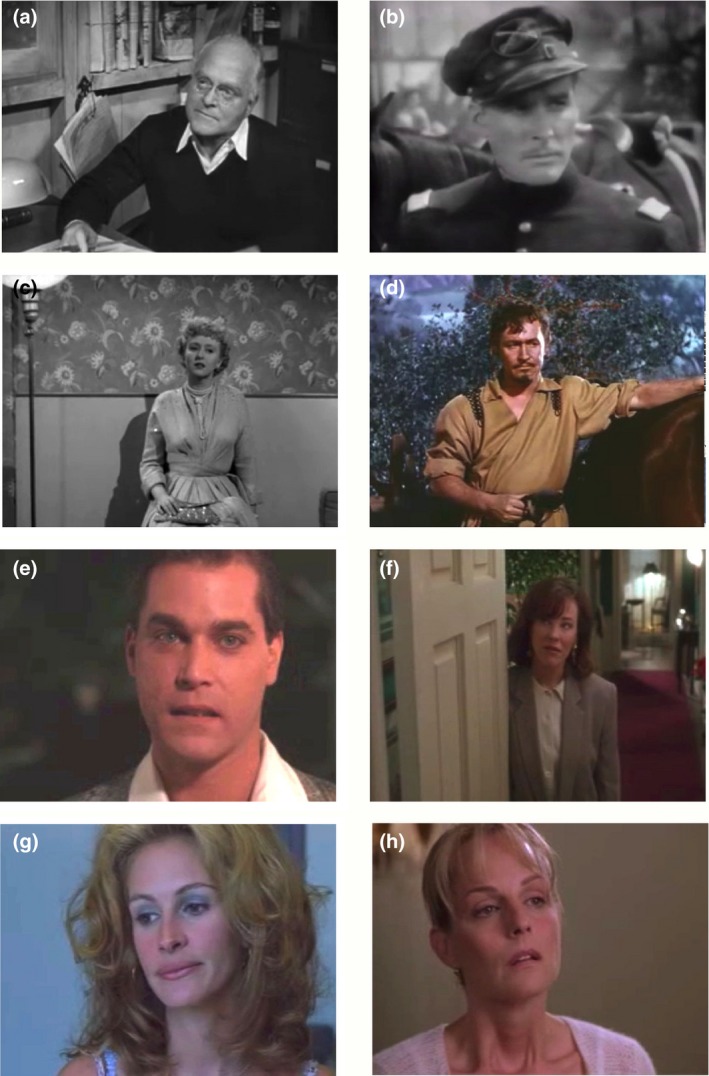
Stills taken from conversation‐ending (cryptic) reaction shots in eight movies: (a) the migrant camp caretaker (Grant Mitchell) in *The Grapes of Wrath* (1940); (b) Jeb Stuart (Errol Flynn) in *Santa Fe Trail* (1940); (c) Karen Richards (Celeste Holm) in *All About Eve* (1950); (d) the Marchese de Granazia (Robert Douglas) in *The Flame and the Arrow* (1950); (e) Henry Hill (Ray Liotta) in *Goodfellas* (1990); (f) Kate McCallister (Catherine O'Hara) in *Home Alone* (1990); (g) Erin (Julia Roberts) in *Erin Brockovich* (2000); and (h) Darcy Maguire (Helen Hunt) in *What Women Want (* 2000 *)*. Notice that three of the four older reaction shots (a, c, and d) are medium shots or longer, and that three of the four newer reaction shots (e, g, and h) are close‐ups.

Four classes of stimuli were assembled. For the critical class, we took stills (static frames) of faces from a sample (72 of 300) of the reaction shots at the end of the conversations discussed in Study 2; 28 were from dramas, 26 from action movies, and 18 from comedies, and across the eight decades, we sampled 5, 8, 6, 5, 12, 12, 12, and 12 reaction shots, respectively. All of these came from roughly the middle of the shot and were full face, three‐quarter profile, or somewhere in between. These included shots taken from 22 of the 24 movies (one of the other two movies had no cryptic reaction shots), and they never included the same actor more than twice. We call these the *movie* faces (Group A). Eight of these images are shown in Fig. 5, two each from movies released in 1940, 1950, 1990, and 2000. Notice that, consistent with the results of Study 1, three of the four older reaction shots are medium shots or longer, and that three of the four newer reaction shots are close‐ups.

To anchor extremes and midpoints of the scales, we selected three other samples of faces. First, we chose 18 faces from Ekman and Friesen (1975). There were three each for the alleged basic emotions of anger, disgust, fear, sadness, and surprise (Group B, *n *=* *15). We will call these the *ardent* faces. Three other Ekman and Friesen faces represented the basic emotion of happiness, and these are included in a later group. We do not subscribe to a theory of basic emotions, but we felt that the Ekman and Friesen faces would be effective in eliciting extreme judgments. All of these stimuli were full face.

We also felt obligated to focus on the center of the valence scale and the bottom of the arousal scale. So, for a second additional sample, we chose 18 neutral faces of young adults from the online collection associated with Righi, Peissig, and Tarr (2012, http://wiki.cnbc.cmu.edu/Face_Place). We call these the *neutral* faces (Group C). They also allowed us to diversify our population of faces with more Asians and more women; the Hollywood sample is, by historical practice, extremely White and predominantly male, and the Ekman and Friesen sample is almost all White. Online, the Righi et al. faces are three‐quarter profile and face to the right. We flipped half of these to face left.

Finally, we created a third supplementary sample of 18 stimuli through Google image searches for “happy” or “smiling” faces, which we call the *happy* faces. This allowed further racial, gender, and age diversity and populated the larger sample with more high‐valence tokens. There were a few such stimuli in the other sets (three from Ekman and Friesen), and these were added to the Google images (Group D; *n* = 21). Most of these were full face.

All told, there were 72 males and 54 females represented in the stimuli; with 11 Asians, six Blacks, and at least five of mixed race (from the Righi et al. sample); with five older individuals (gray hair or wrinkles showing) and six children. Finally, 39 of the images were in black and white and 87 in color.

### 5.2 Methods

We used a 7‐point scale for each dimension, valence and arousal, with viewers rating each face twice. Four purpose‐written MATLAB scripts and interfaces presented stimuli at mid‐screen to each viewer on a 17‐inch MacBook Pro viewed from about 0.6 m. Stimuli were presented in 4:3 aspect ratio, if necessary trimmed from their original formats or filled in with black side panels if they did not fill this format initially. All images subtended about 8° of visual angle measured vertically.

Two scripts presented random sets of practice trials (eight trials each for valence and arousal judgments) and, for the test phases, two scripts presented individually randomized sequences of the 126 stimuli, once for valence judgments and once for arousal judgments. For the valence judgments, a 1 (very negative) to 7 (very positive) vertically oriented scale appeared on the left‐hand border of each picture. Viewers moved a computer mouse to orient crosshairs over the rating they would choose to give and pressed the mouse for their response. The interface measured responses continuously from 1 through 7 (that is, for example, a response of 4.37 could be recorded). The next trial began immediately after the mouse press. Pauses were allowed every 42 trials, and a computer file of responses was stored at the end of the run.

The second run presented the arousal scale on the right edge of the frame with a similar array of numbers, 1 (very low arousal) to 7 (very high arousal). This test presented the same images in a new random order. Responses were initiated and stored in the same manner. The arousal test was always run after the valence test because it is easier to explain the valence variable, and we wanted viewers to already have experience with the procedure before tackling the slightly more difficult‐to‐explain task of arousal judgments.

For both tasks, we also recorded reaction times, but we did not stress speed in the instructions. We had no firm predictions here, but it has been reported in the literature that positively valenced expressions are responded to faster than negative ones (Leppänen & Hietanen, 2004), a result that our previous research had partly corroborated (Cutting & Armstrong, 2016).

We advertised for 20 subjects, but 21 Cornell undergraduate students showed up so we ran them all (11 females; four Asian, two Hispanic, two Black, and one Arab). All participated for course credit. They were run individually, and the experimental session lasted about 20 min. This study was exempted from a full IRB review.

### 5.3 Results

For the stimuli, there were no effects of gender, race, age, or (for the movie faces) release year on judgments of valence or arousal. The medians of the 210 intercorrelations among viewers’ judgments were *r*s = 0.72 and 0.71, respectively, for valence and arousal responses. Median intercorrelations among their reaction times were nearly zero, and indeed, there were no effects here of reaction time. Given the strong congruence of judgments across observers, all analyses below are on the stimulus items.

**Figure 6:**
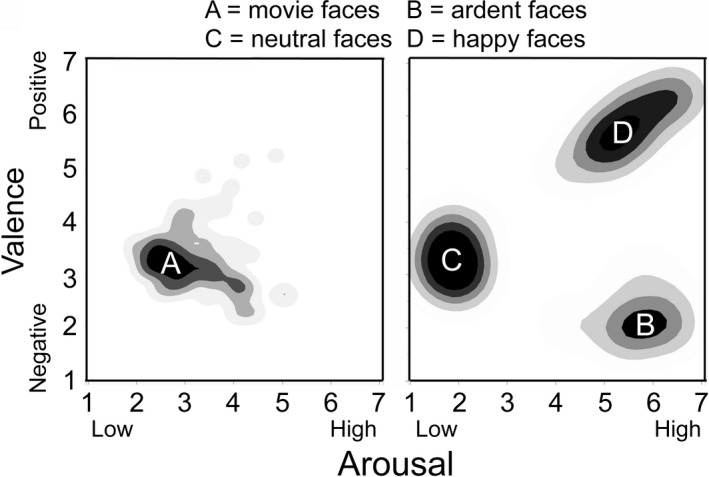
Results of Study 3. Rating data are plotted as density clouds in Cartesian coordinates of valence against arousal. The darkest regions represent the smoothed 20% thresholds of the data, followed by lighter areas of 40%, 60%, and barely visible 80%. The data represented in the left panel (*movies* faces, Group A) represent judgments of stills taken from the cryptic reaction shots (*n *=* *72). The data in the right panel show the three sets of control stimuli; Group B represents the *ardent* faces (three each of anger, disgust, fear, sad, and surprised faces from Ekman & Friesen, 1975; *n *=* *15); Group C represents the *neutral* faces (from Righi et al., 2012; http://wiki.cnbc.cmu.edu/Face_Place; *n *=* *18); and Group D represents the *happy* faces (*n *=* *21, 18 from a Google image search and 3 from Ekman & Friesen, 1975).

Mean viewer judgments of valence and arousal for the 126 stimuli are shown in Fig. 6, plotted in Cartesian coordinates, valence against arousal. The right panel shows the data plotted as density clouds for the judgments concerning the three control sets of stimuli (groups B, C, and D), and the left panel shows the density cloud for the judgments of the stills taken from the cryptic reaction shots (Group A). Three of the four data sets show uncorrelated variation in both dimensions, but the happy faces (Group D) show a strong correlation between valence and arousal (*r *=* *.90, *t*(19) = 9.0, *p *<* *.0001, *d *=* *4.1), a result consistent with Kuppens, Tuerlinckx, Russell, and Barrett (2013). In addition, the neutral faces (Group C) were rated somewhat below the midpoint of the valence scale—3.31 (*t*(17) = −8.8, *p *<* *.0001, *d *=* *3.6)—a finding consistent with previous research on neutral faces (Lee, Kang, Park, Kim, & An, 2008).

Perhaps, the most interesting within‐set result is the relative uniformity of responses for the ardent faces from Ekman and Friesen (1975; Group B). In particular, the anger, disgust, fear, sadness, and surprise faces show very little variation in their judged valence or arousal. In this experimental context with a wide variety of more naturalistic faces, they were all regarded as highly negative and highly aroused—even the sad faces, which were originally designed to be relatively low in arousal. So much for any notion that these stimuli might represent the varied space of facial emotion.

Many differences among the control sets are very robust. Unsurprisingly, the ardent faces were judged as more negative than the neutral faces (groups B vs. C; *t*(31) = 7.5, *p *<* *.0001, *d *=* *2.7) and also much more aroused (*t*(31) = 20.9, *p *<* *.0001, *d *=* *7.5). The ardent faces were also judged as more negative than the happy faces (groups B vs. D; *t*(34) = 21.8, *p *<* *.0001, *d *=* *7.5) but with no difference in arousal. And finally, the happy faces were judged as more positive than the neutral faces (groups C vs. D; *t*(37) = 17.0, *p *<* *.0001, *d *=* *5.7) and also more aroused (*t*(37) = 19.6, *p *<* *.0001, *d *=* *6.4). Thus, we were quite successful in creating disparate sets of stimuli that populate a triangular affect space, which provide contrasts with one another and create a rating framework within which to consider the movie stimuli.

The movie faces occupy a position amidst these extremes, but not at their center. Although the movie faces were not different from ardent faces in their slightly negative valence (groups A vs. B), they were judged as considerably less aroused (*t*(85) = 11.09, *p *<* *.0001, *d *=* *2.4). In addition, although they also did not differ from the neutral stimuli in valence (groups A vs. C), they were judged as more aroused (*t*(88) = 7.78, *p *<* *.0001, *d *=* *1.66). And finally, compared to the happy faces (groups A vs. D), they were judged as considerably more negative (*t*(91) = 16.9, *p *<* *.0001, *d *=* *3.54) and less aroused (*t*(91) = 12.2, *p *<* *.0001, *d *=* *2.55).

Notice that some of the movie faces were judged to be positive and somewhat aroused. Nonetheless, the majority were judged modestly lower than the midpoints of both scales (mean = 3.3 for valence, *t*(71) = 9.6, *p *<* *.0001, *d *=* *2.2; and mean = 3.2 for arousal, *t*(71) = 8.7, *p *<* *.0001, *d *=* *2.0). Thus, accepting the placement of the judgments of these stimuli as near, but not at, the midpoints of both valence and arousal, we would claim that the characters’ countenances can be regarded as cryptic—slightly averse to, or at least questioning, what is going on, and slightly aroused, looking as if they are quashing an urge to speak. This idea is congruent with Zunshine's (2012) notion that the character is struggling to hold in her emotional response.

## 6 General discussion

### 6.1 The Kuleshov “Effect”

By now it should be clear to anyone with at least a passing acquaintance with film history that we have avoided a pertinent topic—the Kuleshov effect (Levaco, 1974; Pudovkin, 1958). This effect is considered to be the archetypal starting point for discussions of montage and the reaction shot. But therein lies trouble. Montage—the juxtaposition of shots to create a new conjoined meaning—can work in feature‐length cinema but likely not for the putative Kuleshov effect. Movies and movie fragments are different things, and facial gestures that are completely neutral may not work the way that Pudovkin suggested. Let us explain.

Lev Kuleshov made several brief (three‐shot) movies using the famed Soviet actor Ivan Mosjoukine. These films no longer exist but one sequence began with a close‐up of the actor with a neutral expression looking offscreen. This was followed by a shot of an open coffin with a deceased child laying in it. The sequence ended with another close‐up of the actor with the same nonexpression, again looking offscreen. Thus, the sequence consisted of two reaction shots with a POV in between. Second and third variants recycled the first and third shots, but spliced in a bowl of soup in one version and an attractive woman lying on a couch in the other, each replacing the shot of the child's corpse in the coffin.

Reports by Vsevolod Pudovkin (1958), a student of Kuleshov, suggested that viewers read the expression on Mosjoukine's face very differently across the three brief films—the first as deeply saddened, the second as intensely famished, and the third as amorously aroused. Indeed, some viewers apparently extolled the virtuosity of the actor. Such is the suggested power of montage. Many filmmakers, including Alfred Hitchcock (Truffaut, 1966, p. 216), have embraced the validity and power of this three‐shot exercise.

Yet the Kuleshov “effect,” if it exists at all in this three‐shot format, does so only under extremely circumscribed conditions (Barratt, Cabak Rédei, Innes‐Ker, & van de Weijer, 2016; Calbi et al., 2017; Mobbs et al., 2006; Prince & Hensley, 1992). Hochberg and Brooks (1996, p. 265) explained why: “Despite Eisenstein's (1949) assertion that two pieces of film of any kind, when placed in juxtaposition, inevitably combine into a new concept of quality, there is no reason to believe that without specific effort at construal by the viewer anything other than a meaningless flight of visual fragments … will be perceived.” In other words, the filmmakers must first win over the viewer to the narrative and the diegetic world before any mind reading will link the separate shots (see also Cutting, 2005). Three brief shots are not enough. As such, the Kuleshov “effect” is not a psychological effect at all, or at least not one across several Western cultures.

Because of this, we had set the canonical Kuleshov effect aside for our earlier discussion. But its shaky status does not undermine the power of the reaction shot, and the importance of a larger context (Calbi et al., 2017). As Barrett (2017), Bering (2002), and Zunshine (2012) have suggested, we *do* read faces in an obligatory manner, easily and sometimes wrongly imputing emotions and intentions—but we have to know and believe the context. In that of a broader cinematic narrative, the viewer accepts the content of a POV shot as relevant to the response of a character, as well as all other shots that precede and follow a reaction shot. Such is the power of montage in continuous narrative.

Finally, consider the results of Study 3 with respect to the putative Kuleshov effect. The implications of the original staging and editing of the Kuleshov short clips are that a completely neutral face could serve to promote the viewers’ mind reading of a character. The results of Study 3 suggest that filmmakers have generally *not* followed Kuleshov's lead. Instead, at least in the context of conversations, they employ slightly negative and slightly aroused facial expressions on their characters, not completely neutral ones. Again, this is likely to convey the sense of a character's discontent combined with her effort to suppress her emotional response, thus providing the viewer with an optimal opportunity to engage in mind reading within the context of a given conversation.

### 6.2 Theory of mind and cinema

The concept of theory of mind is about one's attribution of mental states to others—their beliefs, desires, goals, intentions. People use this faculty both in the real world and while enjoying the narrative arts. Indeed, there is evidence that the participatory use of this faculty in the arts may be beneficial. It is part of our early and continuing emotional education (Barrett, 2017; Mar et al., 2010; Oatley, 1999, 2012). Zunshine (2006, 2012) has presented an articulated view of how this process serves narrative understanding by promoting our enjoyment and mental play—requisite shots of a focal character *contrast* with other shots of the same character and with those of others (they occur only as 17% of all shots in movies, Study 2), they are *transient* (even briefer than other conversation shots, Study 2), and they are *restrained* in their display of emotion (slightly negative and slightly aroused, Study 3).

This clever combination of affective factors prompts the viewer to attend to the emotional state of the character (most often the protagonist) as a conversation concludes. The viewer then seeks to understand the character's emotional response in order to fully empathize with her as she continues on her diegetic journey. This level of viewer engagement is thought to be a requisite for narrative immersion (van Laer, Ruyter, Visconti, & Wetzels, 2014) and critically, as we have demonstrated here, it is the consequence of cinematic design. Filmmakers seem to be maximizing opportunities for viewers to discern the underlying emotional states of characters, creating an affective bridge to the diegetic world.

## 7 Summary and conclusion

With the decreasing size and increasing mobility of cameras over the 20th century, filmmakers enlarged the average image of characters on the movie screen to what seems to be an optimal size—roughly, that of the medium close‐up. This shot scale minimizes the gaze differences across viewers, allowing them to see simultaneously both the eyes and the mouth of the character in a single glance as well as getting auxiliary information about the upper body. Most movie conversations are shown at about this scale. Thus, at this visual size, the viewer is best situated to read quickly the emotional expression of a character (Study 1).

By the middle of the 20th century, filmmakers began to change the style in which they portrayed conversations, which are the central core of almost every popular movie. Increasingly over the next 60 years, conversations ended with what we call a cryptic reaction shot (Study 2). This shot portrays an unspeaking character with a relatively unexceptional emotional countenance—a demeanor of slightly negative valence and modest arousal (Study 3). We claim that this facial expression engages the viewer in a mind‐reading exercise, as she tries to understand the mental state of the character and the implications of that state for the ongoing narrative. We claim further that these trends—increasing shot scale and increasing numbers of cryptic reaction shots—reflect the gradual development of a metatheory of mind in filmmaking. Increasingly, filmmakers have “known” that viewers try to read the minds of characters and they have given viewers important opportunities in the course of the narrative to do so.

Movies are remarkably popular worldwide. It should be no surprise that one of the reasons for this is that the craft of filmmaking bootstraps from a large suite of psychological principles. Because making movies is an artisanal pursuit, the hard‐won discoveries of framing, pacing, staging, and the like have proceeded by trial and error and, like the use of reaction shots, have preceded systematic psychological study. We believe it is well worth going back through the history of film to rediscover the psychological principles on which they are based. These can reinforce our understanding of our behavior and attitudes in everyday life.

## Notes

1 We are well aware of some striking claims made for the benefits of narrative engagement in short‐term settings (Kidd & Castano, 2013; but see Panero et al., 2016). We make no such claim here. Instead, our suggestion is about benefits in the long term.

2 “Actually thought about” is shorthand for what, in a fictional narrative, an author or filmmaker created in the story for the character to reveal or not to reveal about what he or she was thinking.

3 Psychology has a long history with what has been called nonverbal communication (see, e.g., Hecht & Ambady, 1999), which has looked at facial and body gesture as a concomitant to verbal exchange. What we call *mind reading* here is connected to that literature, although most of nonverbal communication research concerns manual gesture. Moreover, our focus is quite narrow in the facial gesture domain. We are only considering (a) acting in movies, (b) the design of one character's immediate nonverbal response to another character's words, (c) and how a third person (the viewer) should interpret that response given that design. In other words, this is artful simulation of real life that has an unknown connection to what happens in reality. Filmmakers and viewers, however, appear to accept this artifice.

4 Zunshine's overall rubric is called *embodied transparency*. We are not entirely happy with the notion of transparency. Although Zunshine notes that viewers are often wrong in their imputation of emotional state in the character, the idea of transparency seems to imply that the viewer can look through the veils of language to see the true state of emotions. This process seems too automatic, almost passive. Instead, we believe that the reaction shots provoke the viewer into being more active in trying to discern the emotional state of the character. It may be automatic, but only because the viewer is actively engaged in the narrative.

5 Seventy percent of Hollywood revenue now comes from overseas (http://www.bbc.com/culture/story/20130620-is-china-hollywoods-future), and the revenue from Hollywood films viewed in China alone is now almost equal to the revenue of those same films viewed in the United States. Nonetheless, the results reported here may not generalize to national cinemas elsewhere.

6 Horror films typically show the reaction shot of a character before the POV of the “monster” for us to savor a bit longer the anticipation of fright (Carroll, 2010). In addition, Branigan (1984, p. 103) called these POV/reaction shot pairs the point/object and point/glance shots.
